# PDMS Nano-Modified Scaffolds for Improvement of Stem Cells Proliferation and Differentiation in Microfluidic Platform

**DOI:** 10.3390/nano10040668

**Published:** 2020-04-02

**Authors:** Hadi Hashemzadeh, Abdollah Allahverdi, Mosslim Sedghi, Zahra Vaezi, Tahereh Tohidi Moghadam, Mario Rothbauer, Michael Bernhard Fischer, Peter Ertl, Hossein Naderi-Manesh

**Affiliations:** 1 Department of Nanobiotechnology, Faculty of Biological Science, Tarbiat Modares University, Tehran 14115-154, Iran; hadi.hashemzadeh@modares.ac.ir (H.H.); t.tohidi@modares.ac.ir (T.T.M.); 2 Department of Biophysics, Faculty of Biological Science, Tarbiat Modares University, Tehran 14115-154, Iran; a-allahverdi@modares.ac.ir(A.A.); mosslimsedghi@gmail.com (M.S.); z.vaezi@modares.ac.ir (Z.V.); 3 Vienna University of Technology, Faculty of Technical Chemistry, Institute of Applied Synthetic Chemistry & Institute of Chemical Technologies and Analytics, Getreidemarkt 9, 1060 Vienna, Austria; mario.rothbauer@tuwien.ac.at; 4 Department of Health Science and Biomedicine, Danube University Krems, 3500 Vienna, Austria; michael.fischer@donau-uni.ac.at

**Keywords:** nanomaterials, nanoparticle-polymer composites, nano biointerface, microfluidics material, stem cells, osteogenic differentiation, PDMS functionalization, nanotopography

## Abstract

Microfluidics cell-based assays require strong cell-substrate adhesion for cell viability, proliferation, and differentiation. The intrinsic properties of PDMS, a commonly used polymer in microfluidics systems, regarding cell-substrate interactions have limited its application for microfluidics cell-based assays. Various attempts by previous researchers, such as chemical modification, plasma-treatment, and protein-coating of PDMS revealed some improvements. These strategies are often reversible, time-consuming, short-lived with either cell aggregates formation, not cost-effective as well as not user- and eco-friendly too. To address these challenges, cell-surface interaction has been tuned by the modification of PDMS doped with different biocompatible nanomaterials. Gold nanowires (AuNWs), superparamagnetic iron oxide nanoparticles (SPIONs), graphene oxide sheets (GO), and graphene quantum dot (GQD) have already been coupled to PDMS as an alternative biomaterial enabling easy and straightforward integration during microfluidic fabrication. The synthesized nanoparticles were characterized by corresponding methods. Physical cues of the nanostructured substrates such as Young’s modulus, surface roughness, and nanotopology have been carried out using atomic force microscopy (AFM). Initial biocompatibility assessment of the nanocomposites using human amniotic mesenchymal stem cells (hAMSCs) showed comparable cell viabilities among all nanostructured PDMS composites. Finally, osteogenic stem cell differentiation demonstrated an improved differentiation rate inside microfluidic devices. The results revealed that the presence of nanomaterials affected a 5- to 10-fold increase in surface roughness. In addition, the results showed enhancement of cell proliferation from 30% (pristine PDMS) to 85% (nano-modified scaffolds containing AuNWs and SPIONs), calcification from 60% (pristine PDMS) to 95% (PDMS/AuNWs), and cell surface marker expression from 40% in PDMS to 77% in SPION- and AuNWs-PDMS scaffolds at 14 day. Our results suggest that nanostructured composites have a very high potential for stem cell studies and future therapies.

## 1. Introduction

PDMS (polydimethylsiloxane) is a widely used polymer in microfluidic devices for cell and tissue engineering and cell-on-a-chip due to its biocompatibility, permeability to gases, transparency, easy molding and economic affordability. Intrinsic drawbacks of PDMS cause the inappropriate surface for long-term cell analysis [1,2,3,4]. Although oxygen plasma treatment, chemical functionalization, and protein coating make the surface biocompatible for cell adhesion, the hydrophilicity reaction caused by oxygen plasma is reversible and also depends on reaction time and temperature. On the other hand, cell attachment and cell aggregations are improved only in areas where the protein has been coated [1,5,6,7]. Given the increasing demand for microfluidics stem cell-based assays, the tailoring of biocompatible and improved biointerfaces to promote cell-surface interactions is highly demanded. 

The requirement for advanced stem cell technology has dramatically increased in the last decade, due to the importance of stem cells for medical therapeutics, drug development, and a variety of health-care applications including toxicological studies, disease modeling, and cell replacement therapies [3,8,9]. Mammalian cell adhesion on the substrate surface is pivotal to measure cell viability, proliferation, and differentiation [1], and cell growing behavior significantly depends on surface chemical properties [6]. Chemical modification of surfaces can potentially improve cell adhesion and long-term culture. Previous attempts to increase cell viability on PDMS substrate using plasma treatment, silanization, and polymer functionalization have shown improved cell behavior, but the effect was not acceptable. In addition, the use of nanomaterials in the case of PDMS functionalization has not been evaluated so far.

Recently, nano-engineered materials have been widely applied in the areas of drug delivery, biosensing, stem cells, biomedical science, nanotoxicity evaluation, and tissue engineering [3,10,11,12,13,14,15,16]. For instance, osteoblast and stem cells cultured on nano-scaffold have long slender morphology on adhesion to neighbor cells; however, the cells cultured in solid scaffold have flat and smooth morphology [17,18,19,20]. The effective parameters of the micro-electro-mechanical system (MEMS) on cell attachment are surface chemistry (functional group, surface charge, and hydrophilicity/ hydrophobicity), surface roughness, and topography, which can affect protein adsorption and cell attachment [21]. Tissue engineering strategies require migration and proliferation of the cells to fully populate the scaffold to regenerate and recover the injured tissue. Most of the cells have to attach on the surface to proliferate, migrate, and differentiate; therefore, cell attachment is the first step in recovery development. The extracellular matrix (ECM) induces cellular attachment, migration, proliferation, and differentiation. Indeed, artificial ECM use in tissue engineering can mimics the natural ECM in the extracellular matrix [6,19,22,23]. Various binding protein-like fibronectin, vitronectin, and laminin are used in nano-scaffold, and protein adsorption in nano-scaffold was improved to become 2–4 times better than solid-walled scaffold due to the increase of surface area [18,19]. In the study by Xue et al., fibronectin and collagen type (I) have been covalently bonded to 3-Aminopropyltriethoxysilane (APTES) via glutaraldehyde cross-linker on SU-8 surface [6]. They showed that alongside the wettability of the matrix, the attachment and proliferation of the cells increased compared with the tissue culture plate (TCP), free fibronectin, and collagen (I). Although this surface treatment method is very effective, the process involves time-consuming intermediate steps and the human error accumulated between each step may potentially lead to batch-to-batch inconsistencies. Furthermore, the various cells have different behavior on the diverse contexts like an increase of proliferation in nano-context compared with TCP [17,19,24]. The formed ECM in the cell grown on the nano-context in comparison to the solid-walled or micro-context has higher cell attachment ability. Cell therapy can regenerate the function of the cured tissue; however, less control on cell surviving, differentiation, and maturation are its limitation factors in order to be used as a powerful therapeutic technique [25]. The engineered scaffold can control the behavior of stem cells and therefore solve the above-mentioned problems. 

The application of nanoparticles (NPs) in regenerative medicine has a very promising future, but NPs can create unpredictable effects on stem cells and also side effects due to small size and penetration ability into the human body [13,14,15,25]. In general, the NPs used in tissue engineering must have characteristics such as biocompatibility, non-toxicity, preservation of physicochemical properties after surface modification, and no effect on stem cell properties and chemical stability in the physiological environment [14,26,27]. On the other hand, using nanocomposite (NPs and polymer) can improve the cell adhesion, proliferation, and differentiation of stem cells and reduce the potential side effects and risks of nanoparticles [28]. It has been shown that the addition of NPs to the scaffold increases the biological regulation of cell behavior. In a study, apatite nanocrystal merged inside polymer fiber to improve tissue regeneration and cell-cell adhesion. The effects of nanocrystal are more important than microcrystal on biological behavior [1]. The mechanism of this phenomenon is still unclear; however, ECM proteins like vitronectin and collagen may easily be absorbed in the substrate in nanoscale compared with microscale [19]. Using a chemical agent like silane to make hydrophilic PDMS may be harmful to cell growth due to its side effects on regenerative medicine and cell therapy. On the other hand, using NPs in cell differentiation may have some toxicity; however, immobilized NPs in the composite have less toxicity and improve cell growth, proliferation, and differentiation [1]. In our previous work, a thin-film layer for microfluidics cell-based assays was fabricated without PDMS functionalization [3]. The nanostructured microfluidics platform was developed to improve human amniotic stem cell differentiation using fibrin/gold nanowires. The result showed that incorporating gold nanowire into fibrin could significantly enhance the differentiation of stem cells to osteo/chondrogenic fates. The synergic role of stiffness and presence of AuNWs have been emphasized in our previous work, where nanotopographical cues created by AuNWs altered the rate of hAMSC differentiation in comparison with the thin-film layer without AuNWs. Nevertheless, PDMS functionalization using nanomaterials has not yet been evaluated for microfluidics cell-based assays. Chuah et al. found that the addition of wettability using polydopamine improves proliferation and differentiation and keeps the multipotency of MSCs [2]. Therefore, hydrophilic condition and amine formation groups on the surface, which makes a biocompatible surface feasible for mechanobiology and cell analysis purposes. Therefore, the availability of these chemical groups in NPs improves the attachment of the cell to the matrix, and proliferation with regard to most of the protein receptors is located at the cell surface. The rougher surface has a larger surface area, and then the cell adhesion improves on the surface in comparison to a smooth surface, which can lead to increase in cell adhesion [3,28,29,30,31]. Many polymeric substrates that are being used in tissue engineering nowadays have no suitable mechanical properties. Thus, in order to improve their mechanical and other properties, NPs, biopolymers, and inorganic fillers have been used to overcome this drawback [28,32]. Therefore, a simple, environmentally safe, and effective surface functionalization strategy is crucial for rendering biocompatible PDMS surfaces for long-term cell investigation on PDMS-based lab-on-a-chip devices.

**Figure 1:**
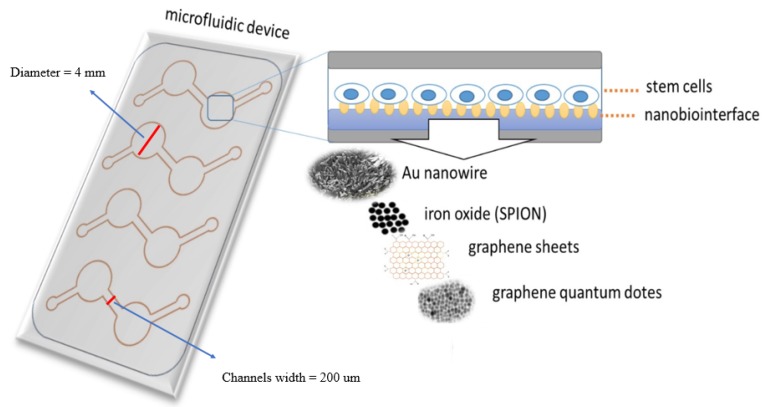
Schematic layout of the microfluidic biochip, experimental setup, and nanomaterials used to synthesize the nanostructured-PDMS (polydimethylsiloxane) composite.

Hence, in the current study, we have doped PDMS with a variety of biocompatible nanomaterials to create a nanomaterial-PDMS composite that presents a biocompatible and tunable nano-biointerface for hAMSC adhesion, toxicity, proliferation, and differentiation. Embedding nanoobjects in the PDMS material thus eliminates tedious surface chemistries and secondary modification steps. In this study, we fabricated and tested various nanomaterial—PDMS composites containing either AuNWs, SPIONs, GO, as well as GQD (see Figure 1). Based on initial cell adhesion studies and surface characterizations, polymer-composite nano-biointerfaces are fabricated and subsequently used to investigate stem cell adhesion, proliferation, and differentiation capacity. 

## 2. Materials and Methods 

### 2.1. Materials 

Gold nanowire (AuNW), graphene oxide (GO), super paramagnetic iron oxide (SPION), and graphene quantum dot (GQD) were synthesized in-house based on our previous research [3,13,15]. Fetal calf serum (FCS) and Dulbecco’s modified Eagle’s medium (DMEM) were obtained from Gibco (all from Gibco, Thermo Fisher Scientific, Waltham, MA, USA) and (3-(4, 5-dimethylthiazol-2-yl)-2, 5-diphenyltetrazolium bromide) tetrazolium assay (MTT) from Sigma Aldrich (Merck KGaA, Darmstadt, Germany). Standard tissue culture grade polystyrene (TCPS) 96-well CELLSTAR^®^ multi-plates (Greiner Bio-One, Germany) were used and PDMS (Sylgard 184) was purchased from Dow Corning Co. Inc. Double distilled deionized water was produced using a Millipore-Q water system and used in all experiments.

### 2.2. Preparation of a PDMS-Based Culture Containing NPs

More details about the synthesis and characterization of NPs are in supplementary materials (S1). Surface modification was performed by immersing the native PDMS surface in six nanoparticles, gold nanowire (AuNW), superparamagnetic iron oxide nanoparticle (SPION), graphene quantum dot (GQD), and graphene oxide (GO). PDMS without any nanomaterials and also tissue culture plate (TCP) were used as a control. Each nanomaterial was mixed with PDMS (elastomer to curing agent 10:1 w/w). The certain amounts of NPs were slowly added to the PDMS under vigorous stirring, spin-coated on glass, and then the mixture was degassed in a vacuum desiccator, incubated at 65 °C for at least 6 h. After complete curing, circles with a radius of 2.1 mm were cut, sterilized for 1–2 h under ultraviolet light, and put in the sterilized condition into a 96-well TCP.

### 2.3. Surface Characterization 

The surface roughness (nanotopography) and Young’s modulus of the PDMS substrates containing SPION, GO and GQD, were determined using atomic force microscopy (AFM, CoreAFM, NanoSurf Co. Ltd, Switzerland and Multi-mode AFM, Ara Research Co., Tehran, Iran) with a tapping mode AFM probe, which comprised a silicon tip with a radius of 28 ± 10 nm and a spring constant range of 0.5–4.4 N m^−1^. 20 μm × 20 μm topographical images were scanned at 0.8 Hz, a set point of 0.7 V, and a resolution of 256 pixels. At least 3 points of contact were analyzed for each PDMS substrate. To measure the roughness of the AuNW/PDMS substrate, SEM images were considered in this case by using ImageJ software (version 1.52i, Fiji, Madison, WI, USA). To describe the roughness of the surfaces, the topography of the surface, and the roughness parameter for the surface, the Sa, which is the area average or the distance between the highest and the lowest point of the surface irregularities, was shown and calculated using built-in software (NanoSurf™ CoreAFM Software, version 3.8.1.4, Switzerland). For Au-NW sample preparation, a drop of water containing Au-NW was placed on an aluminum substrate and incubated until the water evaporated. It was then sputter-coated with a 2 nm layer of gold to prevent charge build-up using a coater and then characterized using a KYKY-EM3200 digital scanning electron microscope (SEM-Model tech, KYKY Technology Development Ltd., Bejing, China). 

### 2.4. Microfluidics Assay 

To create the microfluidics assay, a template for chip fabrication was designed using AUTOCAD 2016 (Autodesk, San Rafael, CA, USA). Then, PDMS sheets were cut using a CAM-1 GS-24 cutter (Roland DGA Corporation, Irvin, CA, USA). Four mm circles from NP/PDMS were cut and used as a substrate in the microfluidic chip (Figure 1). To bind the PDMS-nanomaterial scaffolds and fabricated template to the glass slide, plasma treatment was applied (Harrick Plasma, High Power, 120 s). A certain amount of cells per mL (based on the surface area of the cell culture chamber) were seeded into the microfluidics system, and after expansion, the hAMSCs medium was replaced with osteogenic stem cell differentiation medium (StemMACS OsteoDiff Media, order No. 130-091-678, Miltenyi Biotec GmbH, Bergisch Gladbach, Germany) supplemented with FCS 10% and 1% penicillin/streptomycin. During the stem cell differentiation on-a-chip, the medium was replaced with OsteoDiff Media every 1–2 days, since the chip was kept under CO_2_ (5%), at 37 °C in an incubator. After 7 and 14 days of hAMSCs being treated with OsteoDiff Media, the samples were used to characterize the differentiation rate. 

### 2.5. Immunofluorescence Microscopy and Alizarin Red Staining 

Immunohistochemical staining of the differentiated hAMSCs on NP/PDMS was performed using primary antibodies against collagen type I (Abcam; mouse monoclonal, ab6308, 1/1000 dilution, Cambridge, UK) as follows. The samples were rinsed with PBS several times carefully and slowly, fixated and permeabilized with a solution of 4% paraformaldehyde, 0.5% Triton X100, blocking nonspecific antibodies with 3% BSA, and washed with PBS, using the primary antibodies and wash, blocking nonspecific antibodies with 3% BSA, and washed with PBS. They were then stained with Alexa flour secondary antibody (1/200 dilution monoclonal goat anti-rabbit IgG) and washed with PBS, stained with DAPI (4′, 6-Diamidino-2-phenylindole dihydrochloride). Finally, the stained differentiated hAMSCs cells were viewed using an Olympus IX81 and IX71 (Olympus Ltd, Tokyo, Japan), and the images were analyzed using Fiji software for mean fluorescence intensity (RFU) measurement. For the evaluation of osteogenesis on different NP/PDMS surfaces using alizarin red (2%) (Merck KGaA, Darmstadt, Germany), the medium was removed and each cell chamber was washed several times with PBS and fixed with 4% paraformaldehyde (the paraformaldehyde was heated prior to use to 37 °C in a water bath). Then, the differentiated hAMSCs chamber was immersed in alizarin red, and afterward, to remove the nonspecific staining of alizarin red, the samples were washed gently several times with distilled water (DDW). Finally, an image of the samples was captured using an Olympus IX81 and IX71 microscope and the data were analyzed using Fiji software using optical density measurement. 

### 2.6. Cell Culture Assay

As reported in our previous work [3], human amniotic mesenchymal stem cells (hAMSCs) were used for stem cell differentiation and for investigation of cell adhesion, short-term growth, cell proliferation, cell viability, and cytotoxicity profiles. The study was approved by the ethics committee of the Medical University Vienna (EK791/2008, EK1192/2015), the University Hospital of Lower Austria (GS1-EK-4/312-2015), and the Danube University Krems (Nr.821/2009, 03. Sep. 2015). The placenta was obtained from a healthy delivering woman in accordance with the Austrian Hospital Act (KAG 1982) after written informed consent was signed. The cells were cultured in DMEM medium containing 10% fetal bovine serum and 1% antibiotic solution (Penicillin-Streptomycin) until the desired confluences were reached. Cells were harvested and cultured on NP-PDMS substrates in a 96-well tissue culture plate (TCP) containing DMEM medium containing 10% fetal bovine serum and 1% antibiotic solution [3]. MTT and neutral red assay were used 1, 3, and 5 days after inoculation. To investigate cell behaviors in 1, 3, and 5 days, 4.5 × 10^3^, 3 × 10^3^, and 2 × 10^3^ cells were inoculated on NP-PDMS substrates in the 96-well plate, respectively. Cell adhesion assay was performed via MTT and a neutral red test, following Mosmann [33]. Hence, after cell counting and re-suspending in standard medium, stem cells were inoculated on nanoparticle-containing substrates in a microplate and were placed in the incubator for more than 30 minutes allowing cells to attach. Then the medium was removed gently, to eliminate unattached cells. This procedure was repeated three times for the complete elimination of unattached cells. Then, cells were incubated under CO_2_ (5%), at 37 °C, for 24 h. After reaching confluence the adhered cells were exposed to MTT reagent for 3 h of forming and also neutral red assay. After removing from MTT reagent, dimethyl sulfoxide (DMSO) (Merck KGaA, Darmstadt, Germany) was added to each well and incubated for 15 min on a shaker. Then, the absorbance of the respective wells was measured at 570 nm using a plate reader (Biotek®, Winooski, VT, USA), and the wells filled with only DMSO were taken as blank reference and their amount was subtracted from the other wells. The cell attachment/viability was expressed relative to control values. The same protocol was used for cell viability, cell proliferation, and other behaviors, without washing at the beginning time. 

### 2.7. Statistical Analysis 

After normalizing the collected data, a statistical analysis such as analysis of variance (ANOVA) and mean comparison was performed using statistical analysis software (SAS) version 9.1 (San Diego, CA, USA). Data were subjected to analysis of variance, and means were compared using Tukey’s multiple comparison test with *P =* 0.05. The means ± SE were used to compare the data. The mean optical density (OD) and mean fluorescence intensity (RFU) of each pixel of visualized differentiated hAMSCs were analyzed using Fiji software. For this, more than 800 pixels of each bright field and fluorescence 8-bit images (after converting RGB-image to 8-bit) were subtracted with mean value pixel of background. The average of the resulting values was used for mean comparison of each substrate (NP/PDMS) for both immunofluorescence and alizarin red staining. 

## 3. Results

### 3.1. Nanomaterial Synthesis and Characterization of Nanostructured-PDMS Surfaces

A key concern associated with employing nanomaterials as additives to advance material properties is the quality of the nanomaterial including composition, size, agglomeration status, high surface area, and noble physico-chemical properties [3,12,13,14,34]. To address these concerns the employed gold nanowires, superparamagnetic iron oxide nanoparticles (SPION), graphene sheets, and graphene quantum dots were synthesized in-house and characterized using transmission electron microscope (TEM), Fourier-transform infrared spectroscopy (FTIR), scanning electron microscope (SEM), X-ray diffraction (XRD), and ultraviolet–visible spectroscopy (UV). The UV-Vis spectrum of the AuNWs samples is shown in Figure S1A (Supplementary Data) and features a distinct gold absorption band at 540 nm, which belongs to the transverse plasmon resonance mode. While the longitudinal mode shift beyond the visible area is caused by the micrometer length of the AuNWs, the region of 600–700 nm shows a shoulder, which can be related to nano-disk byproducts. Additional SEM images are shown in Figure S1B (Supplementary Data) and reveal the nanometer size of the rod-like shape of the gold nanowires. The graphene oxide sheets were synthesized as reported in our previous study [35], and more details can be found in supplementary supports (S1). In turn, Figure S1C (Supplementary Data) shows the XRD pattern of the graphene oxide sheets revealing a sharp peak at 2θ = 10.33° corresponding to a spacing of d = 0.935 nm, which is far less than reported for pristine graphene at 2θ = 26.6° corresponding to the diffraction from the (002) plane with a spacing of d = 0.335 nm. The observed shift of the main peak indicates an increase in spacing between the graphene layers due to the incorporation of oxidized surface groups. Additional FTIR spectra of the synthesized graphene oxide sheets are shown in Figure S1D (Supplementary Data). In turn, FTIR spectra of graphene oxide revealed the presence of carboxyl, epoxy, and carbonyl groups in 1726, 1250, and 1050 cm^−1^, respectively. Furthermore the sharp peaks at 3390 cm^−1^ and 1621 cm^−1^ indicate the presence of OH groups, carbonyl groups, and the presence of C=C bonds in graphene oxide sheets [35], while observed peaks at 1219 cm^−1^ and 1027 cm^−1^ indicate the presence of epoxy and alkoxy groups. Finally, Figure S1D (Supplementary Data) shows TEM results from superparamagnetic iron oxide nanoparticles exhibiting an average SPION particle size of around 20 nm. The donated graphene quantum dot results of synthesization and characterization are shown in previous works [35]. Overall, the nanomaterial characterization demonstrates the successful synthesis of the nanomaterial composite. 

**Figure 2:**
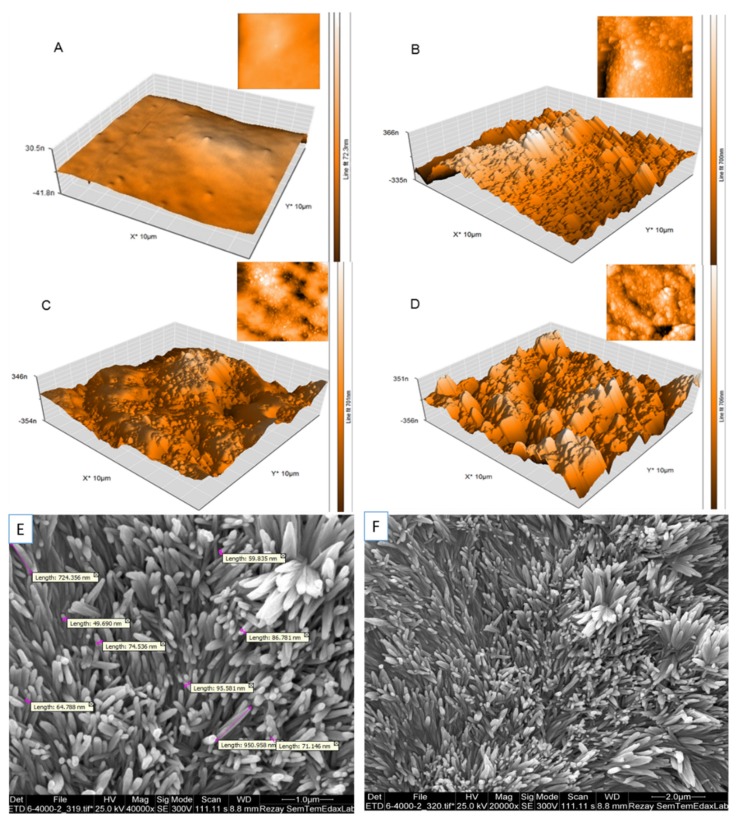
Substratum atomic force microscopy (AFM) topography of unmodified (**A**) and modified PDMS surfaces with nanoparticle coating: GO (**B**), GQD (**C**), SPION (**D**), recorded in tapping mode. The contrast covers height variations in the 0–30 nm scale in A and in the 0–350 nm scale in B, C, and D. Scanning electron microscopy image of the morphology of gold nanowires mixed up with PDMS (**E**,**F**).

**Figure 3:**
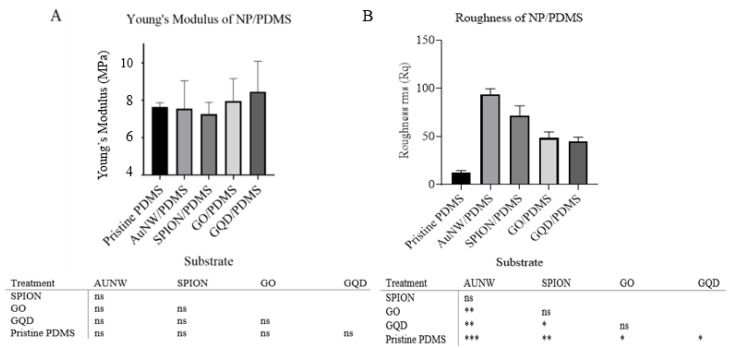
Measured Young’s modulus (**A**) and roughness (**B**) of substrates and the corresponding significance table are presented. ns, *, ** and ***: non-significant, significant at 5%, 1%, and 0.1% levels of probability, respectively.

In the next step, the in-house synthesized and characterized nanomaterials were mixed into the PDMS monomers and polymerized at 70 °C overnight. The precise 300-µm thick PDMS sheets were extensively rinsed with DI water and investigated regarding their surface structures and topography. To assess the impact of surface roughness of the nanomaterial-PDMS interface, AFM and SEM analysis were conducted in subsequent experiments. It is known that a higher roughness profile could provide a higher surface area for the cell–substrate interaction and thus encourages cell adhesion and proliferation. Figure 2 shows 2D (insets) and 3D AFM and SEM images of native PDMS and graphene oxide, graphene quantum dots, AuNWs, and SPION containing nanomaterial-PDMS composite surfaces. Results of the AFM and SEM study demonstrated a 5-fold to 10-fold increase in surface roughness in the presence of the graphene oxide sheets, graphene quantum dots, AuNWs, and SPION-based PDMS composites. The increase from a root-mean-square (RMS) roughness of 14 ± 0.14 nm in the case of PDMS to 97 ± 0.68 nm for PDMS composites points at nanostructured surfaces in the presence of AuNW nanomaterials (highest). In our study, the effect of nanoparticle doped in PDMS on roughness and Young’s modulus and their synergic effects on hAMSC adhesion, toxicity, proliferation, and osteogenic differentiation of hAMSCs were investigated. Based on our findings, no significant difference in Young’s modulus was observed among the substrates containing the nanoparticles and pristine PDMS. Whereas, doping the nanoparticles in PDMS could influence the roughness of the substrates (Figure 3A,B). 

### 3.2. Initital Human Amniotic Mesenchymal Stem Cells (hAMSCs) Viability Assessment 

**Figure 4:**
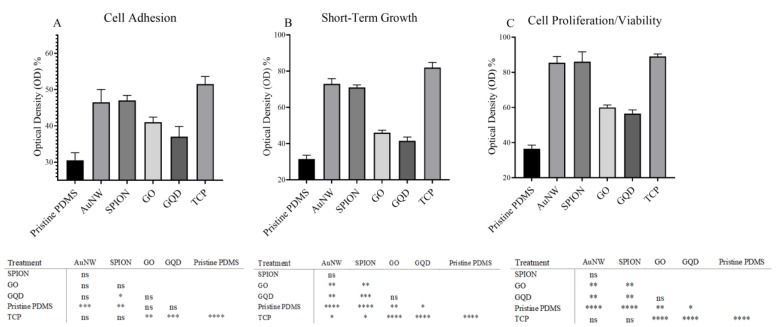
Cell adhesion (**A**) short-term growth (**B**) and cell proliferation (**C**) of substrates and the corresponding significance table is presented. ns, *, **, *** and ****: non-significant, significant at 5%, 1%, 0.1%, and 0.01% levels of probability, respectively. TCP is tissue culture plate.

Following the physical characterization of the nanomaterial content and physical evaluation of the nanomaterial-PDMS composite surfaces, an initial biological study was conducted to assess the ability of cells to adhere, spread, and proliferate on nanomaterial-PDMS composites. In this comparative study, cell morphology and viability of stem cells (hAMSCs) cultures in standard tissue culture well plates on top of pristine PDMS sheets and nanomaterial-PDMS composites were investigated using phase-contrast microscopy, using MTT and neutral red viability tests (the data of the neutral red study are not shown). Due to the range of different conditions including six biointerfaces containing different nanomaterials, ANOVA analyses, as initial evaluations, were performed to determine whether the presence of nanomaterials significantly influences cell behavior over a cultivation period of 5 days, as shown in Figure 4. Already after the 1^st^ day in culture, the mean square of the ANOVA analysis revealed a significant probability at 0.01 level, showing that cell behavior is different between hAMSCs cultured on different nanocomposite substrates. Similar to the first day, a strong impact of various NP surface doping on cell proliferation and viability was observed on the third day, while high cell viability was found over the entire 5 days of exposure to various nanostructured nanomaterial-PDMS composite surfaces. For instance, ANOVA analysis of results obtained from neutral red assays at day 5 showed a significant impact on hAMSCs, thus indicating strong biology-material interactions in the presence of nanostructured surfaces (data not shown). To investigate which of the nanomaterial–PDMS composite surfaces promotes the strongest in cell adhesion, proliferation, and viability, a Tukey’s multiple comparison test was used. Figure 4 shows the cellular behaviors 6 h post-seeding (day 1) to assess cell adhesion, after 3 days to evaluate cell spreading along the biointerfaces, and after 5 days to investigate the influence of surface structure and composition on cell proliferation. Based on the mean comparison for day 1, hAMSCs revealed the strongest cell-to-surface interaction in the presence of superparamagnetic iron oxide and AuNWs nanostructured PDMS surface, while pristine PDMS and GQD-PDMS composites showed weakest cell adhesion, and there was no significant difference among them. Similarly, AuNW and iron oxide nanoparticle–PDMS composite revealed the best stem cell spreading and viability profile at day 3 and day 5 of cultivation. Less strong but still good cell-substrate interactions are found for graphene oxide and graphene quantum dots-modified PDMS surfaces. Interestingly, SPION and AuNW-based PDMS composite surfaces showed higher cell viabilities than pristine PDMS over a culture period of 5 days. In addition, neutral red and MTT results were analyzed to identify the possible effects on cell cultures in more detail. Looking into initial cell adhesion (at day 1), AuNWs and SPIONs-modified PDMS surfaces yielded the best results, while graphene oxide sheets-based PDMS composite, followed by SPION and gold nanowires, exhibited the highest viability and cell spreading after 3 days in culture. Similar to the above results, gold nanowires-based PDMS composite materials yielded optimum cell culture interfaces over a 5-day growth period, followed by SPION. The result of GQD and GO-modified PDMS composites were strongest compared to pristine PDMS. However, no significant difference was observed in comparison to AuNWs and SPIONs-based PDMS in terms of cell behaviors. Overall, these results demonstrate improved cell adhesion, proliferation, and viability profiles of MSC when using SPION and AuNW modified PDMS biointerfaces.

### 3.3. Microfluidics Stem Cell Differentiation Using Nanobiointerfaces

As osteogenic differentiation characteristics, nano-fibrillar proteins like collagen type I is found in bone structure, which serves as a highly organized pattern for calcium deposition [36]. Type I collagen and calcium deposition have been used as osteogenic differentiation biomarkers in different studies [37,38]. 

**Figure 5:**
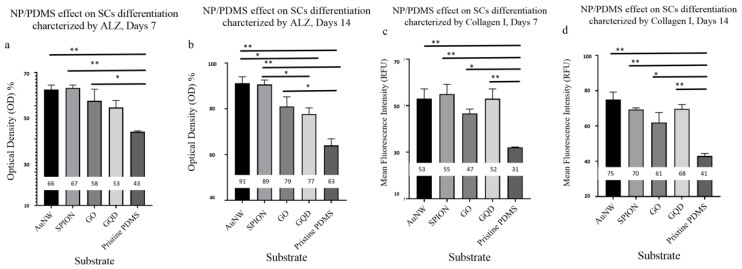
Normalized optical density measurement of calcium deposition of hAMSCs differentiated to osteogenic lineage on nanoparticle (NP)/PDMS substrate (**a**,**b**). Mean fluorescence intensity measurement of immunofluorescence staining of Coll I of hAMSCs differentiated to osteogenic lineage on NP/PDMS substrates (**c**,**d**).

Based on the above results, SPION-, AuNW-, graphene oxide sheets-, and graphene quantum dots-modified PDMS composites were used to assess the stem cell differentiation capacity in subsequent microfluidic stem cell cultivations. Different morphology of human amniotic mesenchymal stem cells (hAMSCs) on nano-modified PDMS scaffolds resulted as scaffolds modified by nanomaterials (Supplementary materials, Figure S2). The impact of nanostructured biointerfaces on osteogenic stem cell differentiation was evaluated using immunofluorescence staining of collagen I expression and also alizarin red staining of bio-mineralization events (Figure 5). Osteogenic lineage of the commitment of hAMSCs is shown in the supplementary information (Supplementary materials, Figures S3 and S4), where the expressed osteogenic marker (Coll I) and calcium deposition are compared between the different nanomaterial-PDMS composite biointerfaces. In all cases, a significant increase in osteogenic marker expression was found in the presence of nanostructured PDMS surfaces, thus pointing at improved stem cell differentiation (Figure 5). 

The hAMSCs were seeded on the microfluidics chip containing NP/PDMS scaffolds and treated with osteogenic media. Calcium deposition and Coll I expression are osteogenic markers. Based on our results these two markers were found to be significant for the PDMS containing nanoparticles. Microchannels containing PDMS/NPs significantly improved the calcium deposition and Coll I surface markers expression in comparison to pristine PDMS (Figure 5 and supplementary materials, Figures S3 and S4). The expression of Coll I was significantly higher in the PDMS/NPs than in the pristine PDMS, suggesting that the presence of nanoparticle mixed with PDMS somehow has a combined effect on osteogenic stem cell differentiation. In addition, the calcium deposition was evaluated and the same results were found in this study. These results clearly suggest that there was a correlation between the nanoparticles embedded into PDMS and osteogenic stem cell differentiation.

## 4. Discussion

The current study was carried out to evaluate nanostructured biointerface fabricated using PDMS/NPs composites for stem cell viability and differentiation. Stem cell viability, osteogenic stem cell differentiation, and bone regeneration are largely affected by physical and chemical cues in the surrounding microenvironment [3,34,39]. In this study, it was shown that nanostructures containing SPION, AuNWs, GO, and GQD nanoparticles, due to high surface to volume ratio and their nanoscale size, can promote hAMSCs adhesion, proliferation, and differentiation. Microscopic image processing showed cultured hAMSCs have high cell density on the cell substrate. Moreover, the cell morphology of hAMSCs cultured on the nano-biointerface is different in comparison with the morphology of cells cultured on pristine PDMS (Supplementary Materials Figure S2). Regarding initial cell viability on the nanostructured biointerface, the substrates containing AuNWs and SPION nanomaterials showed strong cell-substrate interactions. Enhancement of cell proliferation from 30% (pristine PDMS) to 85% (nano-modified scaffolds containing AuNWs and SPIONs) resulted in altering the surface properties of PDMS. In addition, in the case of cell adhesion, nano-modified PDMS with AuNWs and SPIONs had a 46%–47% increase, while pristine PDMS had a 30% increase. Furthermore, the short-term growth rate of hAMSCs on pristine PDMS was 30%, while this rate raised to 74% and more on AuNWs/SPIONs-modified PDMS. This effect can be explained by the fact that pristine PDMS without chemical modifications is a flat and very hydrophobic biointerface, which reduces protein adsorption, which is involved in cell-substrate interactions, and thus reduces proper cell adhesion and spreading. The synergic effects in surface chemistry, topology, stiffness, roughness, and mechanical properties were observed by using various nanoparticles. In this study, the rate of calcification increased from 60% (pristine PDMS) to 95% (PDMS/AuNWs), and cell surface marker expression from 40% in PDMS to 77% in SPION- and AuNWs-PDMS scaffolds at 14 days. Since PDMS-GQD and PDMS-GO substrates when exposed to fluorescence light have intrinsic fluorescence emission, the Coll I biomarkers expression (compared to alizarin red results) could not be well evaluated for the hAMSCs differentiated on GO and GQD/PDMS at the point of analysis, but calcification assays confirmed its positive effects on hAMSCs differentiation. For this, we suggest to carry out more verification for stem cell differentiation using qRT-PCR or immunofluorescence antibodies staining with different emission in the range of emission of these nanoparticles. The results of previous studies showed that the nano-topographical properties of the scaffold are more important than microscale ones in osteogenesis. Moreover, cell responses will be affected by the disorder degree of nanopatterns, while stem cell fate could be determined by the dimension and orientation of nanopatterns. Integrin-related focal adhesion formation along with the reorganization of cytoskeleton are the most important cell functions affected by roughness [3,11,34,39]. Hence, in this study cell substrates with nanoscale properties seemed to facilitate protein adsorption in the process of initial cell viability, as well as affecting the cytoskeletal changes of hAMSCs and enhancing osteogenesis. Numerous studies have demonstrated that enhanced osteogenic differentiation could be achieved by increasing the roughness profile of a substrate, reducing the surface hydrophobicity, providing functional groups that facilitate surface protein attachment, and subjecting MSCs to nano-topographical cues [3,34,40]. The results showed that the impact of roughness is more important than Young’s modulus, as increased hAMSCs adhesion, proliferation, Coll I expression and calcium deposition were found in hAMSCs cultured on corresponding NP-PDMS substrates. In the case of substrate roughness, Young’s modulus and also the findings of our study indicate that incorporating nanoparticles in PDMS could affect the osteogenic differentiation of hAMSCs through modulating the roughness of substrates. Our study offers beneficial information for the fabrication and development of a nanomaterial-based PDMS platform for different stem cell-based assays and biomedical applications such as tissue engineering scaffolds, drug discovery, disease modeling, and toxicological studies.

As hypothesized, the nanotopography created by the nanoparticle plays an important role in stem cell behavior. The AuNWs and SPION nanostructured surfaces showed the best compatibility conditions for adhesion, proliferation, and differentiation of stem cells. Thus, the nanobiointerfaces prepared in this work can be considered as interesting interfaces which are able to support not only stem cells, but also different cell types. More aspects of the chemical groups of the surfaces, different stiffness, and the properties of nanobiointerfaces and potential biomedical applications of these nanostructured surfaces need to be examined in future works. However, the initial results obtained here including cell viability, mechanical properties, and cell differentiation suggest that this nano-biointerface can be a potential candidate to find applications in on-chip cell-based assays, tissue engineering, and drug screening.

## 5. Conclusions

Surface modification of PDMS with different nanomaterials was investigated in the current study to enhance the biocompatibility of poly (dimethylsiloxane) substrates for hAMSCs adhesion, proliferation, and differentiation. Although adding the nanomaterials could not increase the hydrophilicity of PDMS, the results have shown it induces the optimal enhancement of hAMSCs adhesion, proliferation, and differentiation. Here, we demonstrated that doping of polymer PDMS with nanomaterials can eliminate the adverse effects of the plain polymer surface by offering increased surface roughness (a 5- to 10-fold increase), protein adsorption, and functional surface groups. Increasing the surface roughness (nanotopographical cues) seems to be more important for modulating the stem cells behavior like SCs adhesion, proliferation, and differentiation. While reduced toxicities and improved cell proliferation as well as differentiation of conjugated NP-matrix biointerfaces have already been demonstrated [6], we show, for the first time, that embedding nanoobjects into a standard polymeric material can be used to generate improved interfaces for microfluidic cell culture application. The enhancement of cell proliferation from 30% (pristine PDMS) to 85% (nano-modified scaffolds containing AuNWs and SPIONs), calcification from 60% (pristine PDMS) to 95% (PDMS/AuNWs), and cell surface marker expression from 40% in PDMS to 77% in SPION- and AuNWs-PDMS scaffolds at 14 days shows the potential of nano-modified scaffolds. Furthermore, as we used different nanomaterials doped in PDMS, and different nanomaterials can have different chemical groups and different applications (i.e., gold nanoparticle is used for surface-enhanced plasmon resonance biosensing), our substrate can be useful for biochips with more applications like biosensing. Finally, making these simple, straightforward, and rapid nano-biointerfaces is applicable to open PDMS substrates as well as enclosed PDMS-based microfluidic systems, which can be widely applied in many in vitro studies of stem cell research and microfluidics cell-based assays.

## Supplementary Materials

The following are available online at https://www.mdpi.com/2079-4991/10/4/668/s1, S1: protocol of synthesis and characterization of the nanoparticles including AuNWs, SPION, GO, GQD, Figure S1: Nanoparticle characterization, Figure S2: different hAMSCs morphology on nano-modified substrates, Figure S3: Fluorescence images are immunohistochemical staining using Coll I antibody on the surface of AuNW, SPION, GO, GQD and pristine PDMS substrates; Figure S4: The optical microscopic images of calcium deposition (by alizarin red staining) of hAMSCs differentiated to osteogenic lineage on PDMS nanocomposite substrates.
