# Supplementary material for: PDMS Nano-Modified Scaffolds for Improvement of Stem Cells Proliferation and Differentiation in Microfluidic Platform
Source: Nanomaterials (Basel). 2020 Apr 2;10(4):668. doi: 10.3390/nano10040668 (PMC7221996; doi:10.3390/nano10040668)
Supplement: Supplementary file 1 [file nanomaterials-10-00668-s001.pdf]

nanomaterials

Article

PDMS Nano-Modified Scaffolds for Improvement of Stem Cells Proliferation and Differentiation in Microfluidic Platform

Hadi Hashemzadeh [1], Abdollah Allahverdi [2], Mosslim Sedghi [2], Zahra Vaezi [2], Tahereh Tohidi Moghadam [1], Mario Rothbauer [3], Michael Bernhard Fischer [4], Peter Ertl [3,*] and Hossein Naderi-Manesh [1,2,*]

1 Department of Nanobiotechnology, Faculty of Biological Science, Tarbiat Modares University, PO Box: 14115-154, Tehran, Iran; Hashemzadeh.hadi@gmail.com (H.H.); t.tohidi@modares.ac.ir (T.T.M.)
2 Department of Biophysics, Faculty of Biological Science, Tarbiat Modares University, PO Box: 14115-154, Tehran, Iran; aallahverdi@gmail.com(A.A.); mosslimsedghi@gmail.com (M.S.); m_v1382@yahoo.com (Z.V.)
3 Vienna University of Technology, Faculty of Technical Chemistry, Institute of Applied Synthetic Chemistry & Institute of Chemical Technologies and Analytics, Getreidemarkt 9, 1060 Vienna, Austria; mario.rothbauer@tuwien.ac.at
4 Danube University Krems, Dept. of Health Science and Biomedicine, Vienna, Austria; michael.fischer@donau-uni.ac.at
* Correspondence: naderman@modares.ac.ir (H.N.M.); peter.ertl@tuwien.ac.at (P.E.)

Supplementary Materials

S1: Synthesis and characterization of the nanoparticles

Gold nanowire (AuNWs) synthesis

To synthesis AuNWs, anisotropic elongation method was used [1]. Briefly, to increase the synthesis yield, seed and growth solutions were prepared separately. To prepare seed solution, firstly, sodium citrate (0.25 mM), chloroauric acid ($HAuCl_4$ 0.25 mM) was prepared in 20 mL double distilled water, then 600 µL $NaBH_4$ (0.1 M) was added to the mixture in high-speed rotation. After adding $NaBH_4$ to the solution the color turned into deep red and spherical gold nanoparticle, approximately 4nm diameter was formed which is stable at 4°C for a couple of months. This solution was incubated for several hours at room temperature till $NaBH_4$ evaporated. For the initialization of anisotropic elongation of AuNWs, a growth solution was prepared. To prepare a growth solution, CTAB (0.1 M) was added to 200 mL Deuterium-depleted water (DDW), then $HAuCl_4$ (0.25 mM) and ascorbic acid (0.5 mM) was added to the solution, in this stage, the color of the solution was turned from deep yellow to pale yellow due to ascorbic acid (Au (III) to Au (I) reduction). Finally, nitric acid (70 mM) was added. In order to increase efficiency and reach high aspect-ratio AuNWs, the solution was aliquoted into three glass vials as follows: 25 mL glass vials No 1 and 2 in and 250 mL glass vial No 3. The final solution (growth solution) was aliquoted between glass vial No 1, 2 and 3, therefore glass vial No 1 contains 9 mL, glass vial No 2 contains 18 mL and glass vial No 3 contains 173 mL. Growth of AuNWs was started by adding 1 mL seed solution to glass vial No 1 under vigorous stirring for 15-30 seconds, and then under vigorous stirring 1 mL solution of glass vial No 1 was transferred to glass vial No 2 for 30 seconds. Finally, after 30-60s; under vigorous stirring 5 mL of the solution of glass vial No 2 was transferred to glass vial No 3, and kept for 2 hours under stirring at 37˚C. The solution collected in the centrifuge tube (50 mL) and left for 1 week without stirring at 37˚C. The brown pellet formed at the bottom of the centrifuge tube which contains %90 AuNWs is usable and the supernatant was discarded. The pellet was re-suspended in DDW before used.

Graphene oxide (GO) synthesis

Graphene Oxide was synthesized in 85% yield from graphite by the method of Hammer *et al* with slight modification [2]. Briefly, 0.5 g graphite was dissolved in 50 mL cold H_2SO_4 under high stirrer, then 0.5 g $NaNO_3$ powder and 3 g $KMnO_4$ powder were added gently to the mixing solution. Mixing was carried on for 2 hours, then 100 mL DDW was added to the solution, incubated in 98˚C for 3 hours, and cooled down to 60˚C. 3 mL hydrogen peroxide (30% wt) was added, diluted with DDW, incubated in room temperature for overnight. Graphene oxide synthesis yielded (final solution) was filtered by a paper filter (Grade No. 40 filter paper, What man, Kent, UK), washed twice with DDW to remove remains of acidic and then was ultra-sonicate (600 rpm) during 1 hour in DDW. Finally, the solution was centrifuged (20 min) to remove exfoliated graphene oxide.

Super paramagnetic iron oxide nanoparticle (SPION) synthesis

Maghemite (γ-Fe2O3) was synthesized by following Ahmadi *et al* methods [3]. $FeCl_3$ and $FeCl_2.4H_2O$ were dissolved in 2M HCl to reach 2M $FeCl_2.4H_2O$ and 1M $FeCl_3$. Then $NH_3.H_2O$ (2 M) solution was added drop by drop. The mixture kept in room temperature, and the final pH adjusted 9.73. The brown color precipitate was harvested by filtration, rinsed with DDW, and three times Ethanol and dried in 70 C for overnight.

Single layer graphene quantum dots (GQD)

Single layer graphene quantum dot was donated from the department of physics, faculty of basic sciences, Tarbiat Modares University, and all characterizations have been done by Bayat and Saievar-Iranizad. GQD briefly synthesized using glucose (0.05 g) as a precursor in deionized water (50 mL) in a Teflon-lined stainless-steel autoclave (75 mL) by hydrothermal method at 200°C for 8 h [4].

Characterization of nanoparticles

All the nanoparticle synthesized were characterized by using a transmission electron microscope (TEM), Fourier-transform infrared spectroscopy (FTIR), scanning electron microscope (SEM), X-ray Diffraction (XRD), dynamic light scattering (DLS), and Ultraviolet–visible spectroscopy (UV). The characterized data are shown in Figure S2.

Based on the UV-Vis spectrum of AuNWs samples., the absorption spectra of AuNWs is characterized by the absorption band at 540 nm which belongs to the transverse plasmon resonance mode. In addition, due to the micrometers length of the AuNWs, the longitudinal mode has shifted beyond the visible areas in the longer wavelength region. The region of 600-700 nm has a shoulder which related to nano-disk by-products. SEM image of AuNWs indicated that they have a nanometer scale. [1]. The XRD pattern of GO. Pristine graphite gives a peak at 2θ = 26.6°, corresponding to the diffraction from the (002) plane with a spacing of d = 0.335 nm. In the XRD spectrum of GO, however, the most prominent feature is a sharp peak at 2θ = 10.33°corresponding to a spacing of d = 0.935 nm. This shifting of the main peak indicates the increase of the spacing between the graphene layers due to the incorporation of oxidizing groups on them. The steric interactions due to the presence of functional groups are responsible for keeping the GO sheets well-separated, with an increased spacing between them compared to the pristine graphene sheets [5]. The FTIR spectrum of G and the prepared GO sample are shown in Figure S2. Graphite exhibited an obvious peak at 3420 cm^{-1} assigned to the stretching vibration of the hydroxyl group, and another clear peak ~1031 cm^{-1} corresponds to the C—O stretching vibrations. The FTIR spectrum of GO clearly shows the presence of carboxyl, epoxy, and carbonyl groups at 1726, 1250, and 1050 cm^{-1}, respectively. It is apparent that a sharp intense peak at 3390 cm^{-1} is due to the presence of OH groups [6], a sharp peak at 1621 cm^{-1} indicates the presence of carbonyl groups as well as the presence of C=C bonds in graphene oxide sheets. The appearance of peaks at 1219 and 1027 cm^{-1} indicates the presence of epoxy and alkoxy groups. Thus, the FTIR absorption signals from these functional groups confirmed their presence in the prepared material, and by inference, the successful preparation of GO.

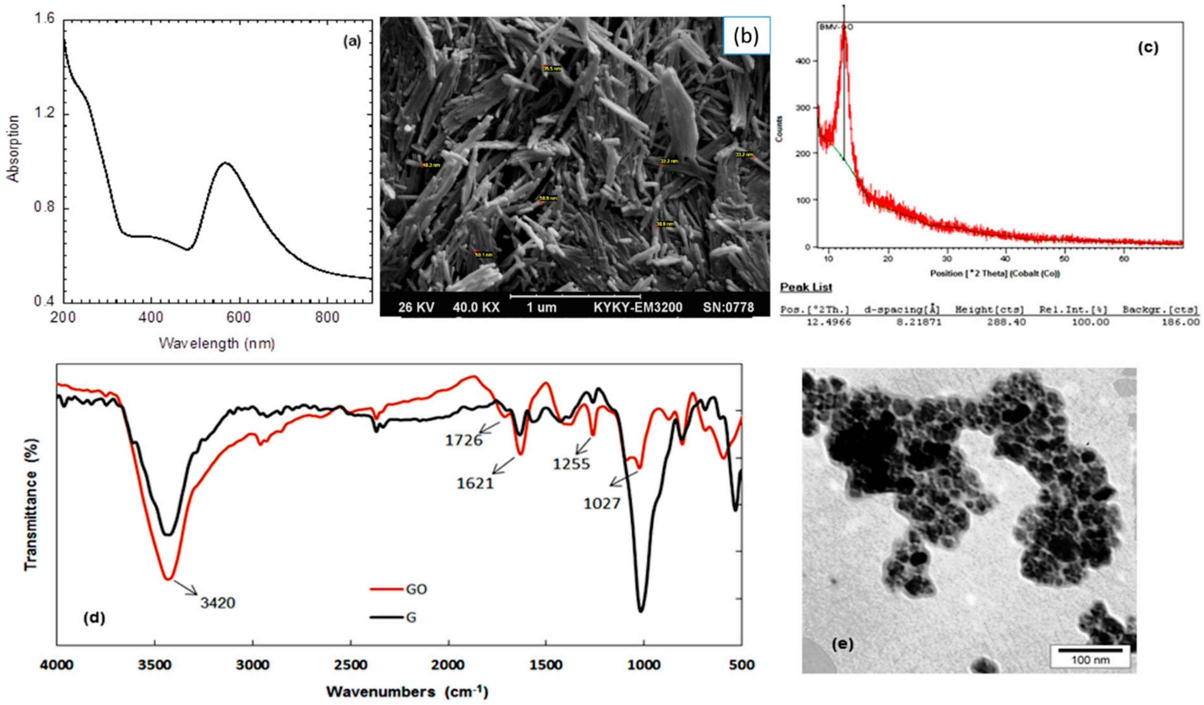

Figure S1. Nanoparticle characterization. (a) UV-Vis a spectrum of gold nanowire sample, (b) scanning electron microscope (SEM) of the gold nanowire, (c) XRD pattern of GO, (d) FTIR of graphene oxide sheets, (e) Transmission electron microscope (TEM) of a SPION nanoparticle of about 20 nm in diameter showing agglomerates property.

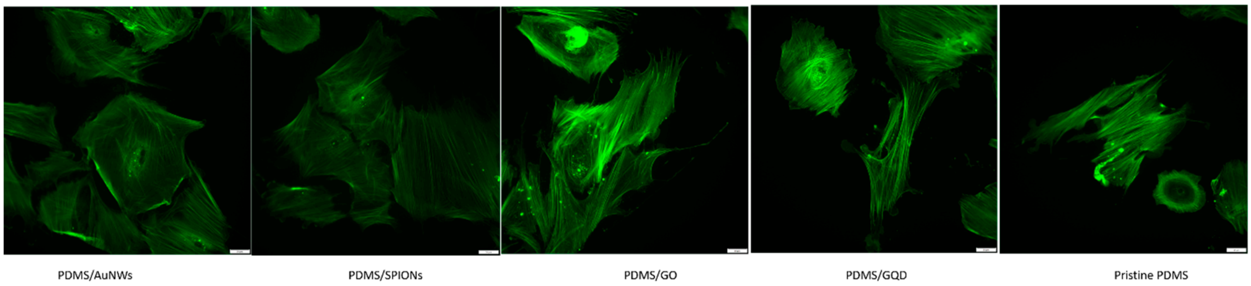

Figure S2. Different morphology of hAMSCs on nano-modified PDMS scaffolds.

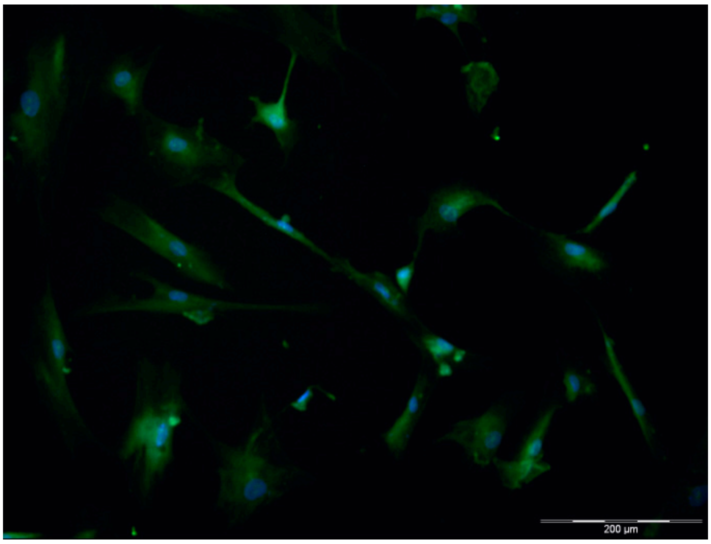

S3-a: AuNWs-modified PDMS

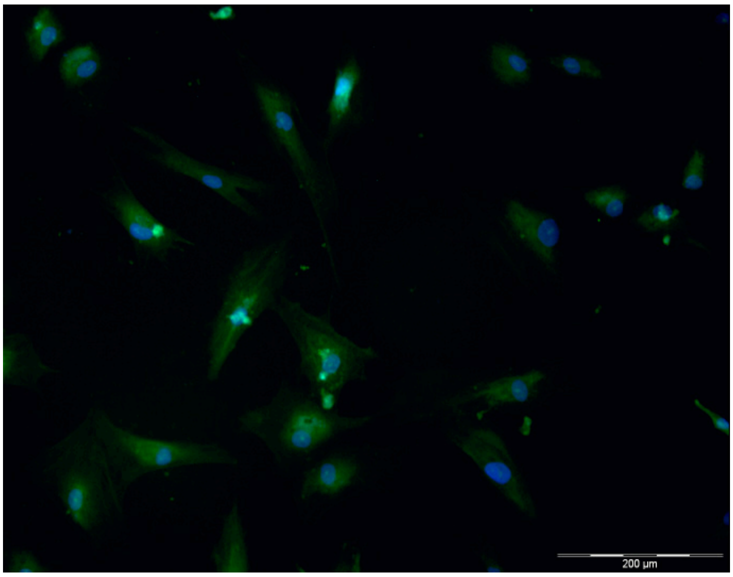

S3-b: SPION-modified PDMS

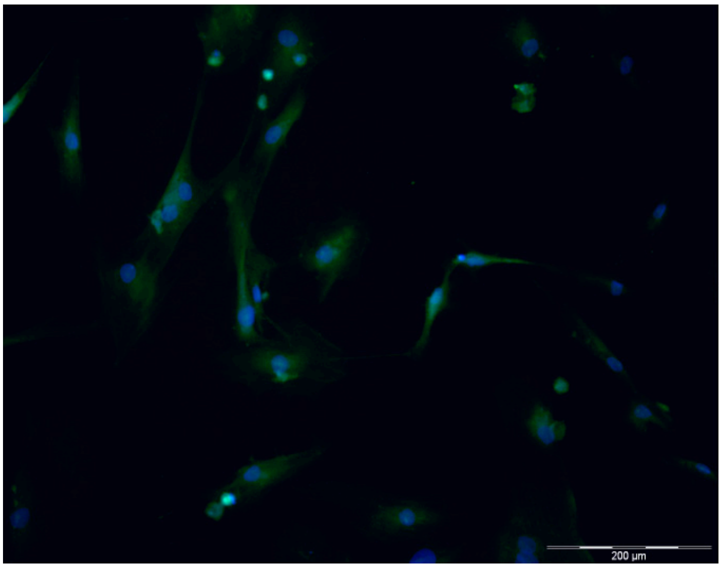

S3-c: Graphene Oxide nanosheet-modified PDMS

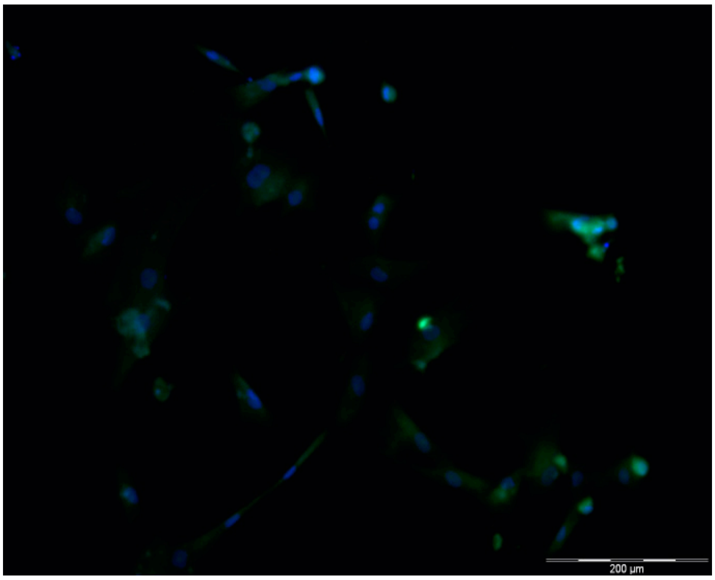

S3-d: Graphene Quantum dot-modified PDMS

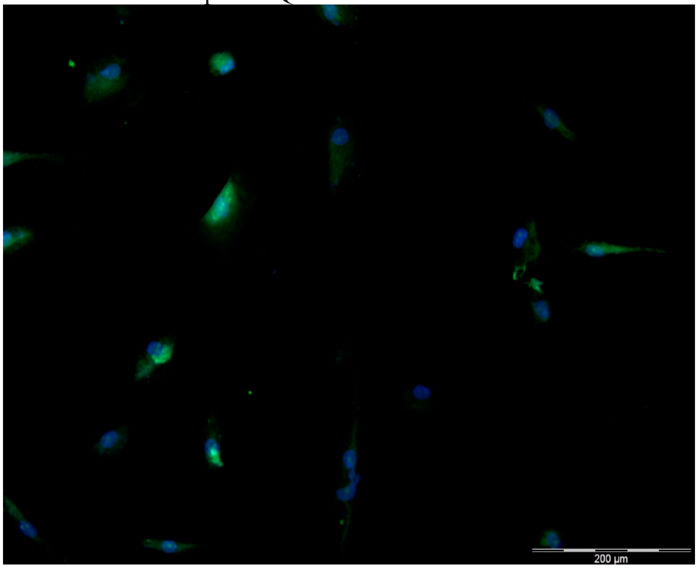

S3-e: Unmodified PDMS

Figure S3. fluorescence images are immunohistochemical staining using Coll I antibody on the surface of AuNW, SPION, GO, GQD and pristine PDMS substrates (Scale bar= 200 μm).

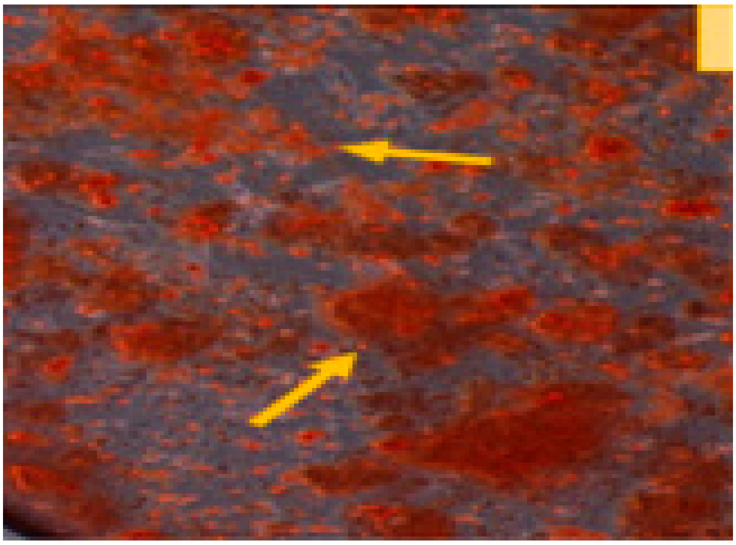

S4-a: AuNWs-modified PDMS

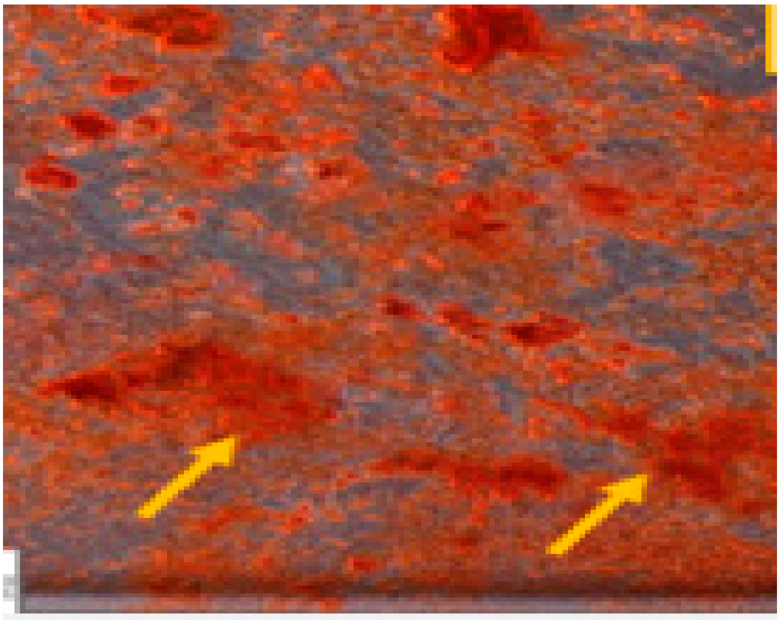

S4-b: SPION-modified PDMS

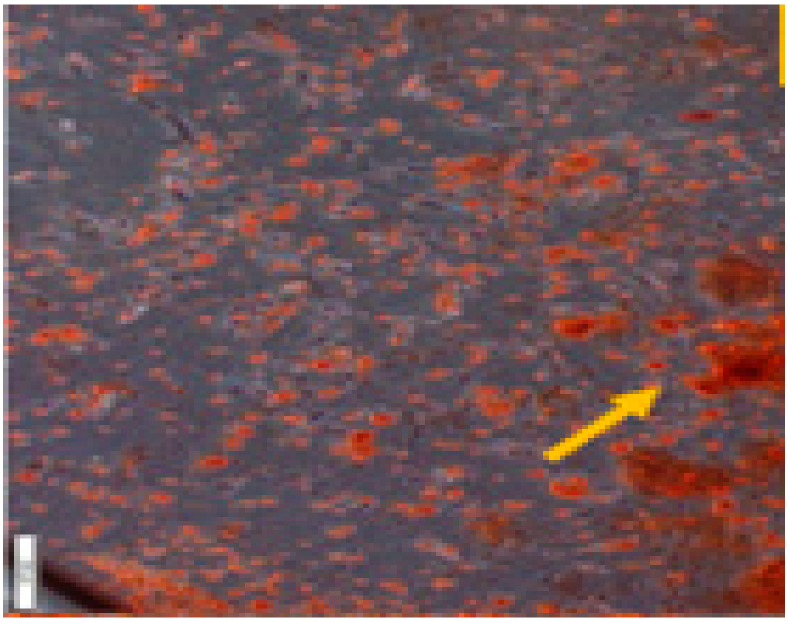

S4-c: Graphene Oxide nanosheet-modified PDMS

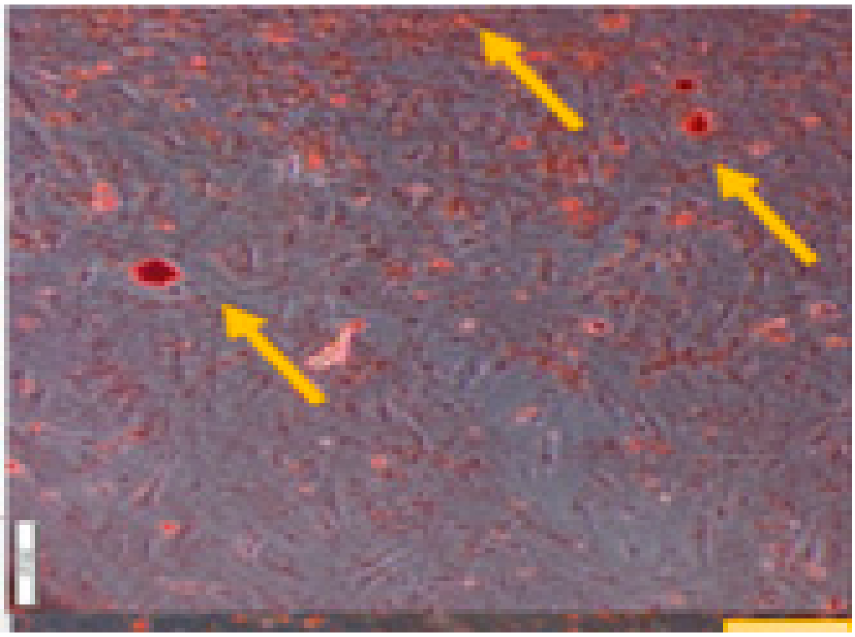

S4-d: Graphene Quantum dot-modified PDMS

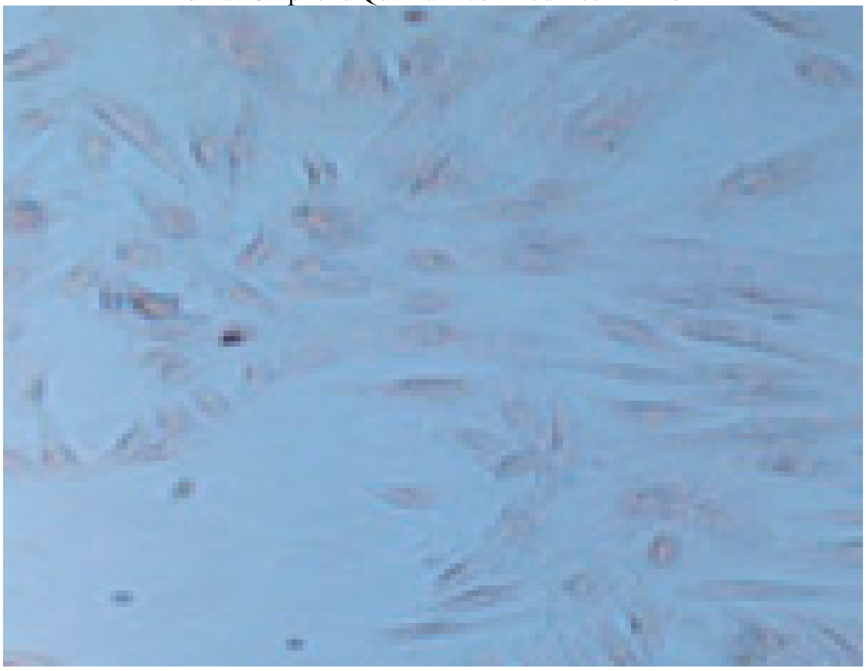

S4-e: Unmodified PDMS (scale bar 100 μm)

Figure S4. The optical microscopic images of calcium deposition (by alizarin red staining) of hAMSCs differentiated to osteogenic lineage on PDMS nanocomposite substrates. Scale bars = 200 μm.

References

1. Kim, F.; Sohn, K.; Wu, J.; Huang, J., Chemical synthesis of gold nanowires in acidic solutions. *Journal of the American Chemical Society* **2008,** *130* (44), 14442-14443.

2. Mazaheri, M.; Akhavan, O.; Simchi, A., Flexible bactericidal graphene oxide–chitosan layers for stem cell proliferation. *Applied Surface Science* **2014,** *301*, 456-462.

3. Ahmadi, A.; Heidarzadeh, S.; Mokhtari, A. R.; Darezereshki, E.; Harouni, H. A., Optimization of heavy metal removal from aqueous solutions by maghemite (γ-Fe 2 O 3) nanoparticles using response surface methodology. *Journal of Geochemical Exploration* **2014,** *147*, 151-158.

4. Bayat, A.; Saievar-Iranizad, E., Synthesis of green-photoluminescent single layer graphene quantum dots: Determination of HOMO and LUMO energy states. *Journal of Luminescence* **2017,** *192*, 180-183.
5. Bhattacharyya, A.; Mondal, D.; Roy, I.; Sarkar, G.; Saha, N. R.; Rana, D.; Ghosh, T. K.; Mandal, D.; Chakraborty, M.; Chattopadhyay, D., Studies of the kinetics and mechanism of the removal process of proflavine dye through adsorption by graphene oxide. *Journal of Molecular Liquids* **2017,** *230*, 696-704.
6. Thakur, S.; Karak, N., Green reduction of graphene oxide by aqueous phytoextracts. *Carbon* **2012,** *50* (14), 5331-5339.

© 2020 by the authors. Submitted for possible open access publication under the terms and conditions of the Creative Commons Attribution (CC BY) license (http://creativecommons.org/licenses/by/4.0/).
